# The Genetic Diversity of *Mesodinium* and Associated Cryptophytes

**DOI:** 10.3389/fmicb.2016.02017

**Published:** 2016-12-20

**Authors:** Matthew D. Johnson, David J. Beaudoin, Aitor Laza-Martinez, Sonya T. Dyhrman, Elizabeth Fensin, Senjie Lin, Aaron Merculief, Satoshi Nagai, Mayza Pompeu, Outi Setälä, Diane K. Stoecker

**Affiliations:** ^1^Biology Department, Woods Hole Oceanographic Institution, Woods HoleMA, USA; ^2^Department of Plant Biology and Ecology, University of the Basque CountryLeioa, Spain; ^3^Department of Earth and Environmental Science, Lamont Doherty Earth Observatory, Columbia University, PalisadesNY, USA; ^4^North Carolina Division of Water Quality, RaleighNC, USA; ^5^Marine Sciences, University of Connecticut, GrotonCT, USA; ^6^IGAP Coordinator, St. George Traditional Council, St. George IslandAK, USA; ^7^National Research Institute of Fisheries Science, Japan Fisheries Research and Education AgencyYokohama, Japan; ^8^Departamento de Oceanografia Biológica, Instituto Oceanográfico da USP, University of São PauloSão Paulo, Brazil; ^9^SYKE Marine Research CentreHelsinki, Finland; ^10^Horn Point Laboratory, University of Maryland Center for Environmental Science, CambridgeMD, USA

**Keywords:** *Mesodinium*, *Teleaulax*, cryptophytes, ciliates, acquired phototrophy, mixotrophy, red tides, ciliate genetic diversity

## Abstract

Ciliates from the genus *Mesodinium* are globally distributed in marine and freshwater ecosystems and may possess either heterotrophic or mixotrophic nutritional modes. Members of the *Mesodinium major/rubrum* species complex photosynthesize by sequestering and maintaining organelles from cryptophyte prey, and under certain conditions form periodic or recurrent blooms (= red tides). Here, we present an analysis of the genetic diversity of *Mesodinium* and cryptophyte populations from 10 environmental samples (eight globally dispersed habitats including five *Mesodinium* blooms), using group-specific primers for *Mesodinium* partial 18S, ITS, and partial 28S rRNA genes as well as cryptophyte large subunit RuBisCO genes (*rbcL*). In addition, 22 new cryptophyte and four new *M. rubrum* cultures were used to extract DNA and sequence *rbcL* and 18S-ITS-28S genes, respectively, in order to provide a stronger phylogenetic context for our environmental sequences. Bloom samples were analyzed from coastal Brazil, Chile, two Northeastern locations in the United States, and the Pribilof Islands within the Bering Sea. Additionally, samples were also analyzed from the Baltic and Barents Seas and coastal California under non-bloom conditions. Most blooms were dominated by a single *Mesodinium* genotype, with coastal Brazil and Chile blooms composed of *M. major* and the Eastern USA blooms dominated by *M. rubrum* variant B. Sequences from all four blooms were dominated by *Teleaulax amphioxeia*-like cryptophytes. Non-bloom communities revealed more diverse assemblages of *Mesodinium* spp., including heterotrophic species and the mixotrophic *Mesodinium chamaeleon*. Similarly, cryptophyte diversity was also higher in non-bloom samples. Our results confirm that *Mesodinium* blooms may be caused by *M. major*, as well as multiple variants of *M. rubrum*, and further implicate *T. amphioxeia* as the key cryptophyte species linked to these phenomena in temperate and subtropical regions.

## Introduction

Species belonging to the genus *Mesodinium* are among the most widely distributed and abundant marine ciliates in coastal and estuarine ecosystems (Leppanen and Bruun, 1986; Sanders, 1995; Bell and Laybourn-Parry, 1999). Red water blooms of *M. rubrum*-like ciliates (= *Myrionecta rubra*) have been recorded since Darwin’s journey on the Beagle (Darwin, 1906), and are recurrent features in many coastal ecosystems (Lindholm, 1978; Crawford et al., 1997; Herfort et al., 2011a). While *M. rubrum* has historically been reported as a single species from numerous global locations (Taylor et al., 1971), cryptic diversity has been suspected within the *M. rubrum* morphospecies for some time, due primarily to variability in cell size (Leegaard, 1920; Lindholm, 1985; Rychert, 2004). Previously, only one environmental study of the genetic diversity of the Mesodiniidae has been published, and it focused only on the *Mesodinium rubrum/major* complex within the Columbia River Estuary (Herfort et al., 2011b). Phylogenetic analysis of rRNA genes spanning the internally transcribed spacer region (ITS) have demonstrated that *M. rubrum* is actually a species complex, composed of at least six major clades (Herfort et al., 2011b; Garcia-Cuetos et al., 2012). One of these clades was described as a new species, *M. major*, based on molecular and ultrastructural characteristics, and is larger and has more plastids than *M. rubrum* (Garcia-Cuetos et al., 2012). Additionally, a new mixotrophic species, *M. chameleon*, was described from primarily benthic habitats, with unique cilia/kinetid structures as well as plastid type and organization of cryptophycean organelles (Moestrup et al., 2012).

Cryptophytes are known to be important components of phytoplankton communities in coastal ocean ecosystems, especially in low light, estuarine, or high latitude environments (Buma et al., 1992; Mallin, 1994; Adolf et al., 2006). While little is known regarding temporal or spatial trends of cryptophyte diversity, *Teleaulax, Plagioselmis*, and *Hemiselmis* are commonly encountered in marine environments (Hill et al., 1992; Cerino and Zingone, 2007; Metfies et al., 2010). Despite the well-established connection between *Teleaulax*-like cryptophytes and growth of *M. rubrum* (Gustafson et al., 2000; Johnson and Stoecker, 2005; Smith and Hansen, 2007), relatively few studies have documented their relationship in nature. Single cell PCR of *M. rubrum* from both coastal Japan and the Columbia River Estuary, have revealed predominantly *Teleaulax amphioxeia* plastids (Nishitani et al., 2010; Herfort et al., 2011b).

All photosynthetic *Mesodinium* spp. are thought to harbor only cryptophyte organelles, which they acquire through feeding on free-living prey (Gustafson et al., 2000). When acquiring organelles from cryptophyte prey, *M. rubrum*-like ciliates also retain mitochondria, cytoplasm, and the nucleus (Johnson, 2011). The nucleus, referred to as a kleptokaryon, remains transcriptionally active and appears to facilitate functional control and division of stolen organelles (Johnson et al., 2007; Lasek-Nesselquist et al., 2015). However, relatively few strains of the *M. major/rubrum* complex have been successfully cultured and studied in detail.

*Mesodinium* spp. are well-documented to form blooms in numerous coastal regions, particularly in estuaries (Crawford et al., 1997; Herfort et al., 2011a) and coastal upwelling zones (Ryther, 1967; Packard et al., 1978). Blooms of *Mesodinium* are highly productive (Smith and Barber, 1979) and form dynamic aggregations within the water column (Crawford and Purdie, 1992). *M. rubrum*-like ciliates can form thin layers in stratified surface waters (Sjöqvist and Lindholm, 2011), concentrate around down-welling frontal regions (Packard et al., 1978), and are capable of self-retention within estuarine systems by vertically migrating to avoid tidal flushing (Crawford and Purdie, 1992). These behaviors are due to *M. rubrum*’s astounding motility, which allow the ciliate to move at speeds of ∼400 body lengths s^-1^ through jumping (Fenchel and Hansen, 2006). One of the best-studied recurrent *M. rubrum* bloom locations is within the Columbia River estuary in the Pacific Northwest of the United States. Blooms in this system have been described to occur annually during late summer, and appear to be caused by only one of the five known genotypes of *M. rubrum* found in the estuary (Herfort et al., 2011b).

Here we present an analysis of the genetic diversity of *Mesodinium* and cryptophyte algal communities from both bloom and non-bloom conditions, in order to shed light on which species and variants of each group are associated with the “red tide” phenomenon. We designed new *Mesodinium* primers are capable of amplifying all known species from the Mesodiniidae family, rather than the *M. rubrum/major* complex only (Herfort et al., 2011b). We also designed one new cryptophyte rbcL primer in order to better anneal with major marine groups of these flagellates, since previous studies (Hoef-Emden, 2005) were focused on genera dominant in freshwater as well. This study establishes a framework for assessing the biogeography of Mesodiniidae genetic diversity and provides new insights into the cryptic diversity of these ciliates and cryptophyte algae.

## Materials and Methods

### Collection of Cell Material

Environmental samples analyzed for *Mesodinium* and cryptophyte diversity were opportunistically gathered from various sources. In most cases (BR, NC, Bar-M4, GF-LL3a, GF-XVI, TV), samples were collected from surface water preserved using 5–10% Lugol’s fixative. However, samples were also collected onto a 0.2 μm Sterivex^TM^ filter (EMD Millipore, Billerica, MA USA) (CL) or centrifuged to form a cell pellet (LIS), and kept frozen until analysis. Previous research has shown that Lugol’s preserved material is sufficient for DNA extraction and quantitative (q) PCR assays, and while sensitivity of the assays decreased overtime, positive amplification was still possible for several months of sample storage at room temperature (Bowers et al., 2000). The sample from SGI, however, was unpreserved and shipping of the sample was slow due to the geographical isolation of the collection site. Upon the arrival of the SGI sample it was immediately centrifuged and the pellet was frozen until DNA was extracted.

### Cultures Used for Phylogenetic Analysis

Cryptophyte and *Mesodinium* spp. cultures were grown in the lab for DNA extraction and sequencing of *rbcL* or rRNA gene fragments for phylogenetic comparison with our environmental data. Cryptophyte cultures including *Teleaulax acuta* (SCCAP K-1486), *T. amphioxeia* (CCMP 1170, GCEPO1), *Geminigera cryophila* (CCMP 2564), *Chroomonas* sp. (CCMP 270), *Hemiselmis pacific* (CCMP 706), *H. andersenii* (CCMP 439), *H. rufescens* (CCMP 440), *Hemiselmis* sp. (SUR21-C3), *Hemiselmis* sp. (NR11), and *Hanusia phi* (CCMP 325) were grown in the lab in F/2-Si at 18°C on a 14:10 L:D cycle, as were *M. rubrum* cultures AND-A0711 and CBJR05. Additionally, the *M. rubrum* cultures were maintained with GCEPO1, by feeding the ciliate weekly with a 1–5% volume addition of log-phase cryptophyte culture. A *Falcomonas* sp. culture (CCMP 2293) was also grown on F/2 without added Si (F/2-Si), but at 4°C and constant light. All of the above cryptophyte and *Mesodinium* cultures were harvested for DNA extraction by centrifugation of 10 ml of dense culture within a 15 ml Falcon tube at 4000 RPM for 15 min, the supernatant decanted, and the cell pellet frozen until processed. Cultures of *Teleaulax gracilis* (Cr6EHU), *T. minuta* (Cr8EHU), *T*. cf. *merimbula* (Cr59EHU), aff. *Teleaulax* (Cr22EHU), *Plagioselmis nannoplanctica* (Cr50EHU), *P.* cf. *prolonga* (Cr10EHU, Cr127EHU, Cr143EHU, Cr194EHU) and *Urgorri complanatus* (Cr1EHU) were grown in the same light and temperature conditions described above, on a modified F/2-Si (enriched with soil extract and selenium at 0.006 mM Na_2_SeO_3_ final concentration) at a salinity of 30 (*P. nannoplanctica* strain grown in freshwater), and were preserved in ethanol prior to DNA extraction and analysis of the *rbcL* gene. A live sample of *M. rubrum* culture MR-INO200702 and a 5% Lugol’s-preserved sample of culture MR-MAL01 were also acquired for analysis. For all cultures, approximately 10–20 ml of dense stationary-phase culture was collected by centrifugation (4000 RPM, 10 min) and frozen until extraction.

### DNA Extraction and Gene Fragment Amplification and Sequencing

Nucleic acids of environmental samples were extracted from frozen cells collected by centrifugation (4000 RPM, 10 min) or on Sterivex^TM^ filters (EMD Millipore) using either the PowerWater Sterivex DNA Isolation Kit (MO BIO Laboratories, Inc.) or a hot detergent lysis method as described by Gast et al. (2004), modified to exclude zirconia-silica bead disruption. All cultures as well as the North Carolina Bloom sample were extracted using the Qiagen DNeasy Plant Mini Kit. PCR was conducted using GoTaq (Promega) or GoTaq G2 Hot Start mix in 50 mL reactions, with a final concentration of 2.5 mM MgCl_2_, 200 μM dNTPs, 2.5 U GoTaq Flexi polymerase, and 0.1 μM primers for normal and 0.2 μM for hot start. Primers for *Mesodinium* spp. (**Table 1**) were designed to amplify the majority of the SSU and LSU rRNA genes, and the entire ITS region, resulting in a ∼1880 bp amplicon. Gene fragments of the large subunit of the ribulose-1,5-bisphosphate carboxylase oxygenase gene (*rbcL*) were PCR amplified from DNA extracts using primers targeting cryptophyte plastids. A new primer, crypt_rbcLR2 (5′-CAGTGAATACCACCTGAAGCTA-3′), designed using an alignment of cryptophyte *rbcL* sequences (See Supplementary Data Sheet 1 for accession numbers) was used in combination with L2F (Hoef-Emden et al., 2005). PCR conditions were: 95°C for 5 min followed by 40 cycles of 95°C for 60 s, 55°C for 60 s, and 72°C for 90 s followed by 72°C for 7 min. The genus-specific primers MESO_245F and MESO_28S_R were used to amplify a combined fragment of the *Mesodinium* spp. 18S-ITS-28S genes. PCR conditions were: 95°C for 5 min followed by 40 cycles of 95°C for 60 s, 57°C for 60 s, and 72°C for 90 s followed by 72°C for 7 min. PCR products were visualized by agarose gel electrophoresis and later excised and purified from the gels using the Zymoclean Gel DNA Recovery Kit (Zymo Research). Clone libraries were constructed from gel purified fragments using the pGEM-T Easy Vector in the pGEM-T Easy Vector System II cloning kit (Promega Corporation) according to the manufacturer’s protocol. Selected clones were submitted for Sanger sequencing with a single primer to either Beckman Coulter Genomics (Single Pass Sequencing) or the W. M. Keck Ecological and Evolutionary Genetics Facility at the Marine Biological Laboratory (Woods Hole). Full-length Sanger sequencing of select *Mesodinium* clones were run at Genewiz (Boston) (see Supplementary Data Sheet 1 for accession numbers). Sequences were edited and assembled into contigs using Sequencher (Gene Codes Corporation). We sequenced and analyzed 687 cryptophyte *rbcL* clones (accession numbers in progress) from environmental samples. For *Mesodinium* spp., a total of 903 clones were sequenced (accession numbers in progress) from all stations. Using a sequence similarity criterion of 98 and 99% for cryptophytes and *Mesodinium*, respectively, we constructed independent contigs and generated consensus sequences for use in our global alignment for each sample. This process helped to reduce the number of sequences used in our phylogenetic analyses while maintaining meaningful diversity data.

**Table 1:** Primers designed and used for amplifying the SSU, ITS, and LSU rRNA genes of *Mesodinium* spp. and *rbcL* gene fragments of cryptophytes in this study.

Primer	Sequence	Gene: position (bp)
***Mesodinium* spp. primers**		
MESO245F	CGACTCGACGTCCCG	18S: 246
MESO580R	CGTCCGTAGTCTGTACGTC	18S: 585
MESO1200F	ATTCCGGTAACGAACGAGAC	18S: 1217
MESO1440F	AACTAGGAATGTCTCGTAAGC	18S: 1446
MESO580R	GACGTACAGACTACGGACG	18S: 603
MESO865R	ACCTTCGTCCTTTGTCGCA	18S: 1017
MESO1480R	CTAAACACTCGATCGGTAGG	18S: 1545
MESO28S_R	AGACTTGGATGACTTTTATCACC	28S: 298
**Cryptophyte primers**		
rbcL2F-800	AGGAGGAAWAYATGTCTCAAT CCG	rbcL: 1
Crypt_rbcLR2	CAGTGAATACCACCTGAAGCTA	rbcL: 1185

### Phylogenetic Analysis and Species Assignment

Consensus sequences from assembled contigs were used for separate alignments of *Mesodinium* spp. and cryptophytes, in combination with available sequences in Genbank and sequences generated from cultures in our laboratory (see above). Alignments were constructed using the Clustal X algorithm (Larkin et al., 2007), and refined by eye using MacClade 4.08a (Maddison and Maddison, 2000). All maximum likelihood (ML) phylogenetic trees were executed with PhyML 3.0 (Guindon et al., 2010) using 100 bootstrap replicates and the general time reversible (GTR) substitution model, while estimating the gamma distribution parameter, proportion of invariable sites, and the transition/transversion ratio. Phylogenetic trees were constructed using TreeDyn 198.3 (Chevenet et al., 2006). Assignment of *Mesodinium* and cryptophyte species was determined by their nearest neighbor match of a known species within ML phylogenetic trees.

## Results

### Sample Characteristics

#### Bloom Samples

The St. George Island (SGI; Alaska) bloom in the Bering Sea was observed within the main harbor, creating dense patches of red water (**Table 2**). The presence of *M. rubrum*-like ciliates was confirmed by microscopy at the time of sampling. A massive *Mesodinium* bloom was sampled from Ilha Bela, São Paulo, Brazil (BR) in the South Atlantic, and microscopic images from samples of the bloom confirmed the presence of large cells (not shown). This bloom stretched for over 100 km of the Brazilian coastline, and was visible from space (Carlowicz et al., 2014). A bloom sample was analyzed from the North Atlantic, in Oyster Bay, Long Island Sound (LIS), and was also documented using satellite imagery (Dierssen et al., 2015). This bloom revealed *M. rubrum* to be present at ∼1000 cells ml^-1^. Another North Atlantic red water bloom was sampled from the Outer Banks of North Carolina (NC), revealing *M. rubrum* at 1750 cells ml^-1^. Finally, streaks of red water were sampled in the South Pacific, ∼25 km off the Chilean coast (CL) during an oceanographic cruise. However, samples from this bloom were only quickly collected from the surface for nucleic acid extraction, with no additional documentation.

**Table 2:** Location and description of samples for analysis of *Mesodinium* and cryptophyte community diversity.

Region	Description	Abbrev.	Date	Lat	Long	Sal PSU	Temp °C	Meso^b^ cells ml^-1^	Sample notes^c^
South Atlantic	Ilha Bela, BZ	BR	2/15/14	-23.80	-45.22	-	24	37.5	bloom
North Atlantic	Long Island Sound	LIS	9/24/12	40.91	-73.60	27.9	21.9	1003	bloom; Oyster Bay
North Atlantic	Waves, NC	NC	10/6/08	35.56	-75.44	28.63	22.5	1750	bloom
North Atlantic	Barents Sea; station M4	Bar-M4	6/27/11	74.53	30.11	35.06	4.9	10	60m
Baltic Sea	Gulf of Finland; station LL3a	GF-LL3a	7/8/12	60.07	26.34	5.26^a^	17.4^a^	-	depth integrated
Baltic Sea	Gulf of Finland; station XVI	GF-XVI	7/8/12	60.25	27.25	4.52^a^	17.5^a^	-	depth integrated
Baltic Sea	Tvärminne Zoological Station	TV	7/31/13	59.83	23.25	5.5	18.5	-	Hanko, FI; conc.^d^
South Pacific	Chile Coast	CL	11/23/10	-19.97	-70.72	34.8	19.5	-	bloom
North Pacific	Bering Sea; St. George Is.	SGI	9/15/12	56.57	-169.68	-	6.6	-	bloom
North Pacific	California Current	CC	7/5/13	36.33	-123.14	33.8	12.8	0.5	2m

*^*a*^Measurements from 1 m, while all others at same depth of sample; ^*b*^Due to the patchy distribution of *M. rubrum* and its ability to form thin layers, these concentrations are at best approximation; ^*c*^Sample depths are surface unless indicated otherwise. Depth integrated samples are from 0.5, 3, 5, 7.5, and 10 m; ^*d*^Sample concentrated with a 10 μm net.*

#### Non-bloom Samples

Samples from the Baltic Sea included one coastal Finland (Tvärminne) and two off shore Gulf of Finland samples (**Table 2**). A non-bloom subsurface sample from the Barents Sea (Bar-M4) was also analyzed and was documented to have 10 *Mesodinium* ml^-1^. A sample taken during a research cruise in the North Pacific within the California Current (CC) was also analyzed, and had low levels of *M. rubrum* present (**Table 2**).

#### Mesodiniidae Phylogeny

**FIGURE 1:**
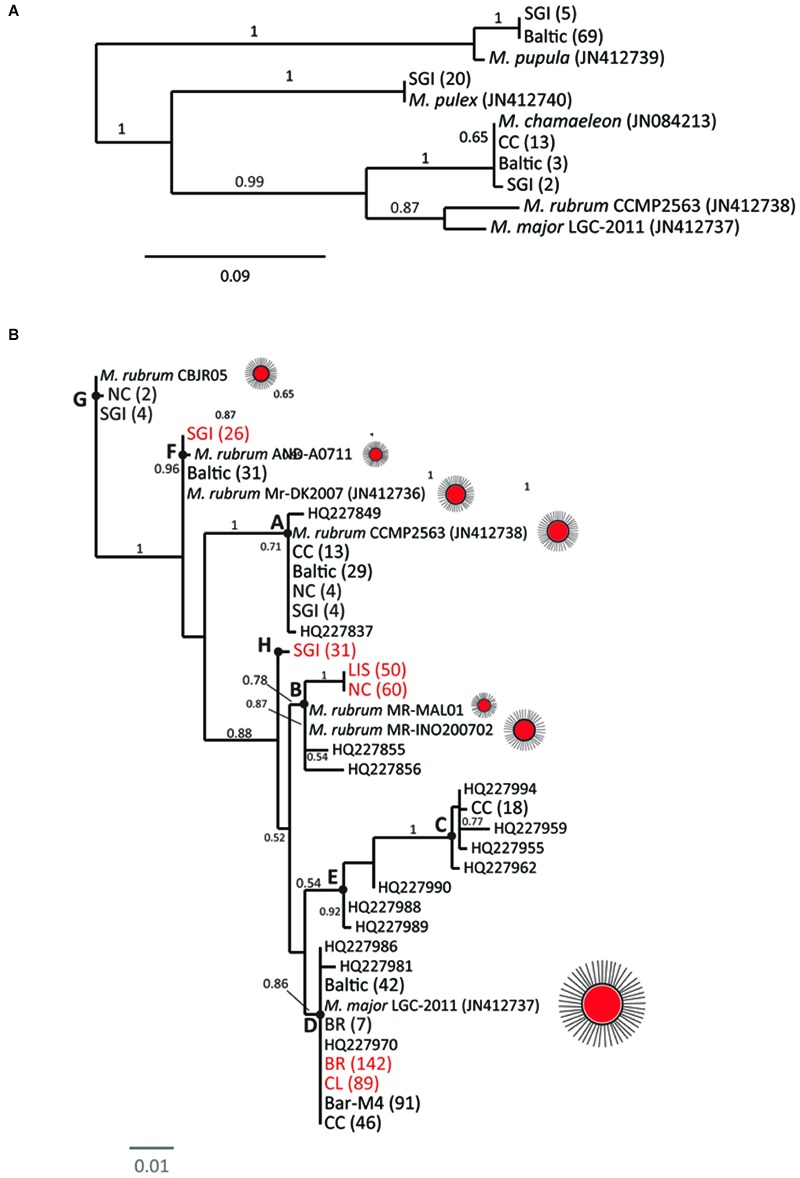
**Maximum likelihood phylogenies of the Mesodiniidae using *Mesodinium* spp. sequences from 10 global locations: the Baltic Sea (stations GF-LL3a, GF-XVI, TV are grouped together in this figure), Barents Sea (Bar-M4), California Current (CC), St George Island (SGI) harbor, coastal Chile (CL), coastal Brazil (BR), Long Island Sound (LIS), and coastal North Carolina (NC).** Phylogeny of the Mesodiniide based on a sequence, composed of a partial SSU rRNA, internally transcribed spacer region (ITS), and a partial region of the LSU rRNA gene. **(A)** Phylogeny of the Mesodiniidae, focusing on community sequences related to *M. chamaeleon* and heterotrophic *Mesodinium* spp. **(B)** Phylogeny of the *M. major/rubrum* complex. Letters on branches of tree (A–H) refer to variants within the complex, and cartoons of the ciliates (oral view) depict the relative cell size of various isolates (**Table 3**). Red text is used for genotypes dominating bloom samples and numbers in parentheses refer to the number of clones representing each genotype.

Using primers designed to amplify a partial region of the 18S, the entire ITS, and a partial region of the 28S ribosomal RNA genes of all known Mesodiniidae taxa, we recovered sequence data from both heterotrophic and plastidic species from eight locations and analyzed their phylogenetic relationships (**Figure 1**). We also analyzed new sequence data from four cultures of *M. rubrum* and one culture of *Mesodinium pulex* (**Table 3**). As shown previously (Garcia-Cuetos et al., 2012) the Mesodiniidae formed four well-supported and distinct clades, represented by *M. pulex, M. pupula, M. chameleon*, and the *M. major/rubrum* complex, respectively, and all comparisons between these major clades revealed the greatest *p*-distance (>7%) within the dataset (**Table 4**). Relatively few environmental sequences were recovered from the *M. pulex, M. pupula*, and *M. chameleon* clades, and intracladal diversity within these groups was lower than within the *M. rubrum/major* complex (**Figure 1A**). Compared to the *M. major/rubrum* variants, the mixotrophic ciliate *Mesodinium chamaeleon* shared 89.6–92% similarity, while *M. pulex* had 83.2–87.7%, and *M. pupula* only 81.2–86% (**Table 4**). *M. chamaeleon* had low similarity to the heterotrophic *M. pulex* (86%) and *M. pupula* (82.9%), as did the latter two species to one another (83.8%).

**Table 3:** Origin and cellular dimensions of *Mesodinium rubrum* variant isolates.

Variant	Strain	Origin/year		Length (μm)	Width (μm)	L/W	Volume^a^ (μm^3^)	Cryptophyte prey	Type reference^h^
A	NCMA 2563	McMurdo Sound, AN 1996	Mean Range	23.1 (2.6) 17.3–30.2	22.4 (2.7) 15.1–32.5	1.1 (0.1) 0.9–1.2	5707 (1826) 2139–10,277	GC^f^	1
B	MR-MAL01	Gomso Bay, KR 2001	Mean Range	21^b^	13	1.6	1857^c^	TA^g^	2
B	MR-INO200702	Inokushi Bay, JP 2007	Mean Range	25.8 (3.8) 16.8–32.1	21.8 (3.8) 13.1–28.6	1.2 (0.1) 0.9–1.6	6719 (3008) 1508–13,007	TA	3
F	AND-A0711	Huelva, ES 2007	Mean Range	16.2 (1.7) 12.6–19.4	13.0 (1.0) 10.1–15.5	1.3 (0.1) 1.1–1.5	1374 (308) 672–1939	TA	4
F	Mr-DK2007	Frederikssund, DK 2007	Mean Range	31^d^ 25–35	21 16–25	1.5	7154^e^ 3349–11,448	TA	5
G	CBJR05	James River, US 2011	Mean Range	21.7 (3.0) 15.8–33.7	16.0 (1.7) 11–21.7	1.4 (0.2) 1.1–2.0	2937 (914) 1234–5537	TA	Here

*All measurements made here unless otherwise noted. Mean values presented with standard deviation in parentheses.*

*^*a*^Volumes calculated as spheroids using 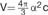 where a is the equatorial radius and c is the polar radius; ^*b*^estimated from Yih et al. (2004); ^*c*^re-calculated here and different from reported value (5996 ± 30) in Yih et al. (2004); ^*d*^from Garcia-Cuetos et al. (2012); ^*e*^calculated here; ^*f*^GC: *Geminigera cryophila*; ^*g*^TA: *Teleaulax amphioxeia*; ^*h*^type references: (1) Gustafson et al. (2000), (2) Yih et al. (2004), (3) Nishitani et al. (2008), (4) Jaén (unpublished), Riobó et al. (2013), (5) Garcia-Cuetos et al. (2012).*

**Table 4:** Genetic p-distance matrix of a partial ‘18S–28S’ rDNA region for variants within the *Mesodinium major/rubrum* complex and other *Mesodinium* spp.

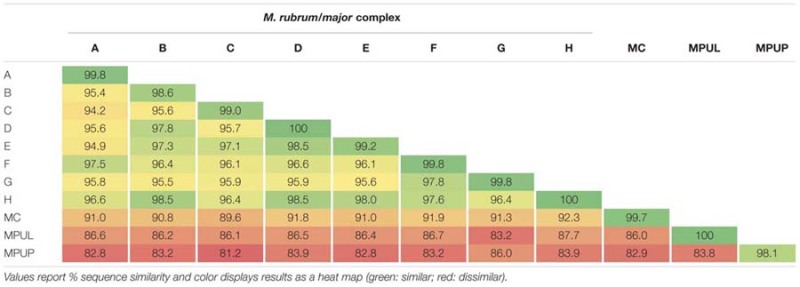

In contrast, the predominantly phototrophic *M. major/rubrum* species complex formed eight subclades (**Figure 1B**) of which six have been described previously (Herfort et al., 2011b; Garcia-Cuetos et al., 2012). The *M. major/rubrum* complex was well-resolved, with four of the clades having 100% bootstrap support within a maximum likelihood phylogeny and three with >75% support (**Figure 1B**). The least resolved clade was that of E, which had both low bootstrap support (54%) and is comprised of only a few environmental sequences from the Columbia River (Herfort et al., 2011b). One of the new clades, designated G, was closely related to clade F, described originally from the Roskilde Fjord in Denmark, and clade A, described originally from McMurdo Sound, Antarctica. Together these three variants form one subgroup within the *M. major/rubrum* complex, while clades B, C, D, E, and H form another. Sequences forming a second new variant, H, were recovered from the SGI bloom sample, and shared high sequence similarity (>98%) with variants B and D (**Table 4**). Lowest intracladal sequence variation was found within variants A, D, F, G, and H (<0.5%), while the greatest variation (>1%) was found within clades C and B (**Table 4**). Polymorphisms between the different *M. major/rubrum* variants are shown in **Table 5** (modified from Herfort et al., 2011b), revealing the sequence variation that shape clade phylogeny within this group. For instance, several shared polymorphisms are found between clades A, F, and G at bases 167, 206, 479, 556, and 569 while clades C and E share four such polymorphisms (bp: 69, 75, 556, and 574). Clades B, D, and H share polymorphisms with each other at bases 191, 212, and 556, and at additional loci with clades C and E at 167, 206, and 479. The most divergent variants within the *M. major/rubrum* group were A and C, sharing 95.7 and 95.9% similarity, respectively, with the other six variants, relative to the average among all groups of 96.6%. Of all *M. major/rubrum* variants, group A had the most autapomorphic characters (10) within the analyzed region, followed by groups G (5) and C (4) (**Table 5**), and these variants were the only to have 100% boot strap support (**Figure 1B**).

**Table 5:** Details of genetic variation for the *Mesodinium major/rubrum* complex within the partial ‘18S–28S’ rDNA region for all available sequence data.

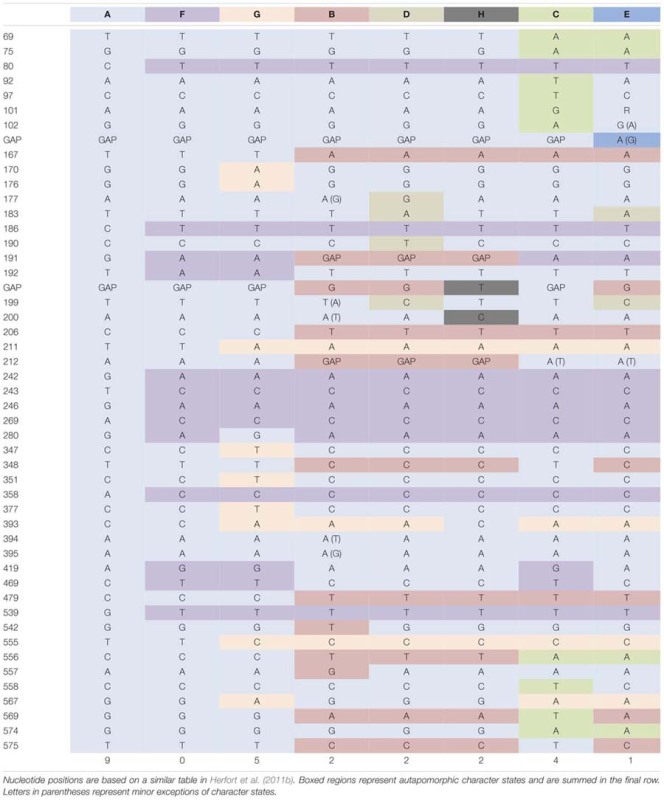

### *Mesodinium* Diversity

**FIGURE 2:**
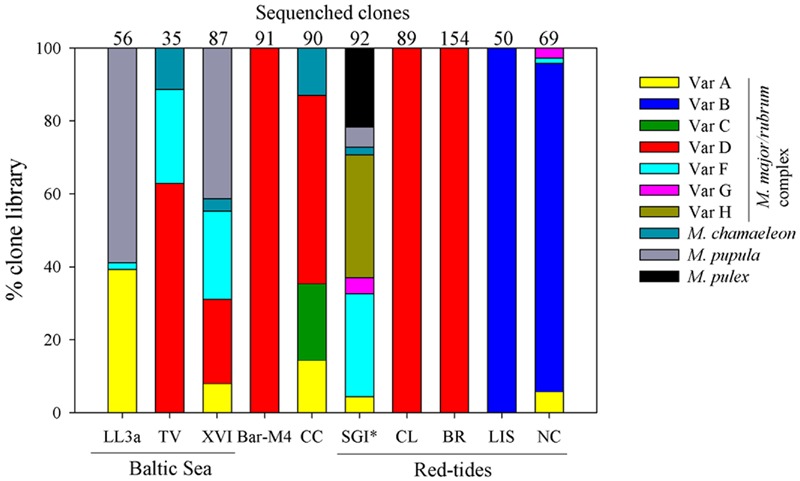
**Community genetic diversity of *Mesodinium* from the Baltic Sea (GF-LL3a, GF-XVI, TV), Barents Sea (Bar-M4), California Current (CC), St George Island (SGI) harbor, coastal Chile (CL), coastal Brazil (BR), Long Island Sound (LIS), and coastal North Carolina (NC).** Community diversity of *Mesodinium* based on a sequence composed of a SSU rRNA gene fragment, the complete internally transcribed spacer region (ITS), and a partial region of the LSU rRNA gene. Categories include seven variants within the *M. major/rubrum* species complex, the mixotrophic *M. chamaeleon*, and the heterotrophic species *M. pupula* and *M. pulex*. ^∗^SGI sample was not properly preserved, so the relative proportion sequences within each category may have been compromised.

The most widely encountered variant was D (*M. major*), found at 6 of the 10 sampling sites, while variants A and F were found at 5 (**Figure 2**). Sequences from variants C and H, as well as the heterotrophic *M. pulex* were each only recovered from one station, while no clade E sequences were found in this study. Analysis of samples from red-water events of *Mesodinium* revealed that the bloom phenomenon within this species complex is not associated with one particular variant. Sequences from subclades B and D dominated (>85%) clones recovered from the four blooms analyzed in this study, and in two cases sequences consisted only of *M. major* (**Figure 2**). Variant B dominated two blooms from the eastern United States, and was not found at non-bloom sites (**Figure 2**). Clones from variants F and H dominated the bloom sample from SGI, however, the sample was not preserved when shipped and thus the relative proportion of *M. major/rubrum* variants is uncertain. Samples from the Baltic Sea were from coastal and open water stations, and were not associated with bloom events. Collectively, the Baltic Sea had the greatest diversity of Mesodiniidae sequences, with clones recovered from heterotrophic *M. pupula*, the mixotrophic *M. chameleon*, as well as from multiple *M. rubrum/major* clades. The CC sample was also diverse, with three variants of *M. major/rubrum* as well as *M. chamaeleon* (**Figure 2**). In contrast, a non-bloom sample from an off shore station in the Barents Sea was composed entirely of *M. major*.

#### Cryptophyte rbcL Phylogeny

**FIGURE 3:**
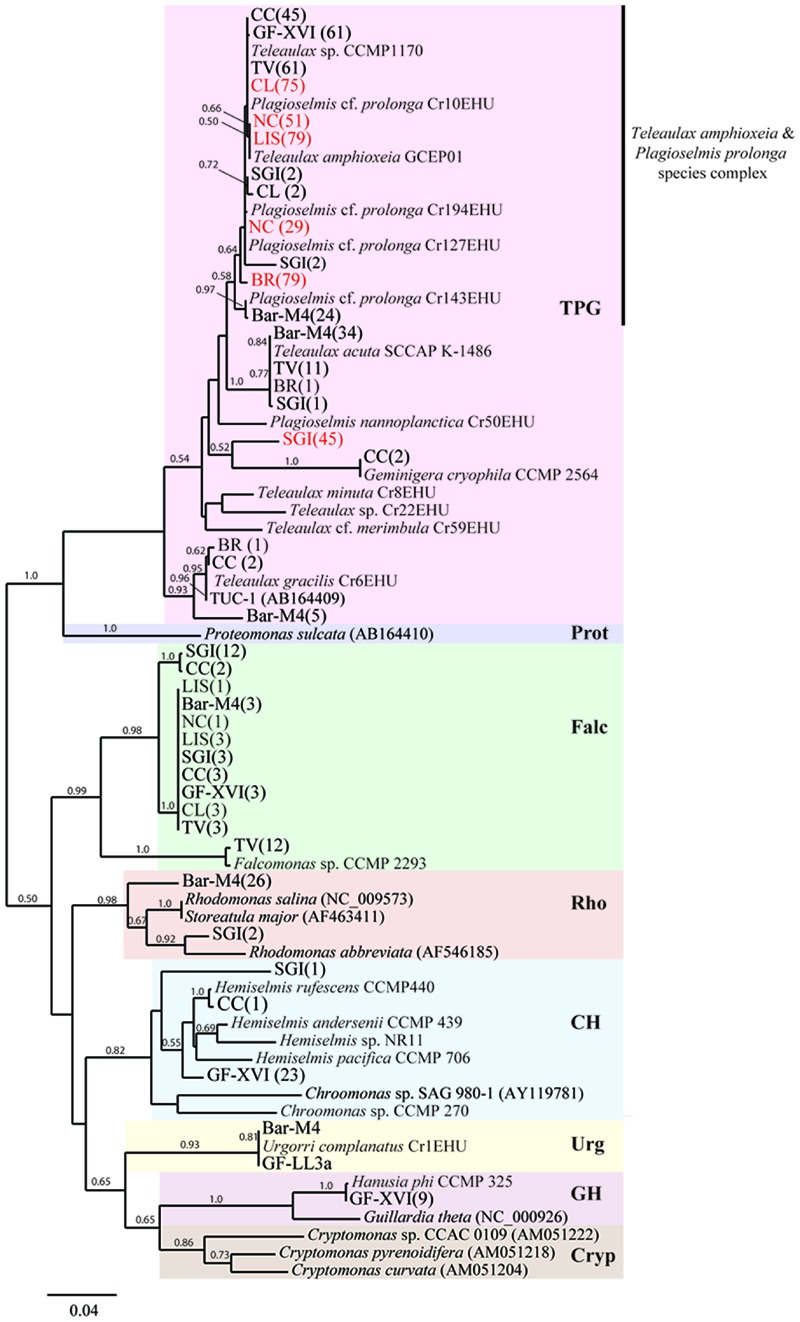
**A maximum likelihood phylogeny of cryptophytes using partial plastid LSU RuBisCO gene (rbcL) sequences from 10 global locations: the Baltic Sea (GF-LL3a, GF-XVI, TV), Barents Sea (Bar-M4), California Current (CC), St George Island (SGI) harbor, coastal Chile (CL), coastal Brazil (BR), Long Island Sound (LIS), and coastal North Carolina (NC).** Red text refers to genotypes dominating bloom samples and numbers in parentheses refer to the number of clones representing a genotype. Major cryptophyte clades are boxed by color: TPG: *Teleaulax*/*Plagioselmis*/*Geminigera* group; Falc: *Falcomonas*-like species; CH: *Chroomonas/Hemiselmis* group; Rho: *Rhodomonas*-like cryptophytes; GH: *Guillardia/Hanusia* group; Urg: *Urgorri*-like cryptophytes.

Our cryptophyte *rbcL* gene data set included new sequences from 22 cryptophyte cultures, comprised largely of TPG and *Hemiselmis* species. Analysis of our environmental clones resulted in 39 distinct contigs, at 99% sequence similarity, from the nine sample sites (one Baltic site was not assessed for cryptophyte *rbcL* diversity). Of these, 21 consensus sequences were determined to be from the *Teleaulax*/*Plagioselmis*/*Geminigera* (TPG) clade (**Figure 3**). Our maximum likelihood tree had strong support (>90 boot strap) for the cryptophyte clades comprised of TPG, *Proteomonas, Rhodomonas*-like cryptophytes, *Guillardia*/*Hanusia, Urgorri*, and *Falcomonas*, and *Cryptomonas*, while the clade comprised of *Hemiselmis* and *Chroomonas* had weaker support (78%) (**Figure 3**). Culture sequences from *T. amphioxeia* and *P.* cf. *prolonga* were polyphyletic, forming a species complex that appears to be in need of taxonomic revision. The position of *Proteomonas* as sister of TPG was strongly supported. No environmental sequences were recovered from the *Cryptomonas* or *Proteomonas* clades. Of all the recovered environmental sequences, those clustering with *Falcomonas* sp. appeared to represent the most novel lineages of uncharacterized cryptophyte species (**Figure 3**).

#### Cryptophyte Plastid rbcL Diversity

Community plastid *rbcL* sequences were analyzed for each sample in order to test the hypotheses that *M. rubrum* blooms are associated with TGP-like cryptophyte sequences and that the number of cryptophyte phylotypes are greater in non-bloom samples. Since these samples are unfractionated community samples, it is not possible to determine the proportion of cryptophyte sequences that are from free-living cells or associated with a particular protist species (e.g., *M. rubrum*). However, while these samples may not specifically represent the diversity of the free-living cryptophyte community *per se*, they are ultimately derived from it. Overall 74% of *rbcL* sequences from all sample sites originated from *T. amphioxeia/P.* cf. *prolonga*-like sequences, while a striking 82% were from the TPG clade. Bloom samples were dominated by TGP cryptophytes (92%), with samples from BR, CL, LIS, and NC sites mostly (>90%) comprised of *T. amphioxeia/P.* cf. *prolonga*-like sequences. In contrast to other bloom samples, the SGI bloom was dominated by a phylotype branching near *G. cryophila*. From non-bloom stations (343 sequences), 56% were *T. amphioxeia/P.* cf. *prolonga*-like sequences, while 72% belonged to the TPG clade. The Bar-M4 sample was primarily composed of *T. amphioxeia/P.* cf. *prolonga*-like sequences, *T. acuta*, and a *Rhodomonas* sp., while *T. amphioxeia/P.* cf. *prolonga*-like sequences dominated the Baltic Sea and CC samples. One of the Baltic Sea sites, station XVI in the Gulf of Finland, was also rich in a *Hemiselmis* sp. and *Hanusia phi*. Sequences from *Falcomonas* spp. were found to comprise a minor component of cryptophyte genetic diversity from eight of the nine sampling sites. *U. complanatus*, which was originally described from Atlantic estuaries in southwestern Europe (Laza-Martínez, 2012), was detected in the Baltic and Barents Seas.

## Discussion

This study is the first to assess the environmental genetic diversity of both non-photosynthetic and plastidic marine *Mesodinium* species, across a broad geographical range of sampling sites. Our findings expand upon the known diversity of the *M. major/rubrum* species complex, and further solidify the relationship of *Teleaulax amphioxeia* cryptophytes with their bloom events. Further, we show novel diversity of cryptophyte algae, particularly among *Falcomonas*-like species, and provide evidence for the widespread dominance of the *Teleaualx/Plagioselmis/Geminigera* (TPG) cryptophyte group.

### Phylogeny of *Mesodinium*

*Mesodinium* spp. have an unusual 18S rRNA gene with numerous deletions and substitutions within conserved regions compared to other ciliates (Johnson et al., 2004), and they appear to lack a separate 5.8S rDNA region, suggesting that it may be fused to the 28S rDNA gene (Herfort et al., 2011b). This characteristic is also found in the microsporidia and bacteria (Hoef-Emden, 2005). While *Mesodinium* show typical litostome secondary structure in their V4 region, including reduction of helices 23_1, 23_8, and 23_9 and the absence of 23_5 (Strüder-Kypke et al., 2006), phylogenies based on the SSU rRNA gene consistently result in a novel, deep basal branch within the ciliates (Johnson et al., 2004; Chen et al., 2015a). From ultrastructural observations, only the ciliary transition region supports their placement within the litostomes (Garcia-Cuetos et al., 2012). A recent multigene phylogeny of the Mesodiniidae also suggests a deep basal ciliate branch and strongly implies that they may represent a distinct ciliate class, the Mesodiniea (Gao et al., 2016). However, a phylogenomic analysis of non-model ciliates using transcriptomic data failed to resolve the monophyly of the Mesodiniea (Chen et al., 2015b).

Our phylogenetic analysis of taxa within the Mesodiniidae agree with Garcia-Cuetos et al. (2012), with strong support for four major clades distinguishing the two heterotrophic lineages of *M. pulex* and *M. pupula*, the mixotrophic *M. chamaeleon*, and the predominantly phototrophic *M. major*/*rubrum* complex. At least one additional major clade of *Mesodinium*-like ciliates are found in freshwater habitats, and phylogenies based on the SSU rRNA gene group them as a sister group to *M. pulex* (Bass et al., 2009). Since we did not sequence samples from freshwater habitats and no cultures of these species were available, comparisons of their full rRNA gene cassette to marine Mesodiniidae were not made here. Our analysis resolved two novel subclades of *M. rubrum*: (1) variant G was identified from sequence data of a culture from Chesapeake Bay, USA, along with environmental sequences from the North Carolina (NC) and St. George Island (SGI) bloom events, and (2) variant H, which is only represented by environmental sequences from the SGI sample. One sequence from a culture isolated in Spain (Riobó et al., 2013) grouped with a Danish culture from the F subclade (Garcia-Cuetos et al., 2012) as well as clones from the Baltic Sea and SGI. Together, variants F and G grouped strongly with variant A to form one major branch of the *M. rubrum/major* complex. We found *M. rubrum* sequences from the Baltic Sea, CC, NC, and SGI that grouped with variant A, which includes a well-studied cultured strain from Antarctica (Gustafson et al., 2000; Johnson et al., 2007) and sequences from coastal Oregon (Herfort et al., 2011b). Cultures isolated from Korea (Yih et al., 2004) and Japan (Nishitani et al., 2008) were found to belong to subclade B, which included sequences from blooms in LIS and NC, as well as the recurrent blooms of the Columbia River Estuary (Herfort et al., 2012). *M. major* (subclade D) was found in the Baltic Sea, Bar-M4, and in blooms from coastal BR and CL, and has also been found in the Columbia River Estuary (Herfort et al., 2011b) as well as coastal Denmark (Garcia-Cuetos et al., 2012). Two other *M. rubrum* variants, C and E, remain uncultured and have only been found in the Northeastern Pacific (here and Herfort et al., 2011b). Subclades B, C, D, E, and H formed the second major branch of the *M. rubrum/major* complex. While C, E, and H have not yet been cultured, subclade B appears to be highly variable in size, both within and between strains (**Table 2**). A bloom of *M. rubrum* variant B in LIS was found to contain cells of typical size for *M. rubrum* cultures (**Tables 2** and **3**).

### Genetic Diversity of *Mesodinium*

The studies of Herfort et al. (2011b) and Garcia-Cuetos et al. (2012) revealed that “*M. rubrum*” is a species complex of closely related genetic variants, most of which are morphologically indistinguishable. While cell size clearly distinguishes *M. major* from *M. rubrum* variants, cell size within *M. rubrum* strains appears to be highly variable considerably (**Table 2**). Furthermore, sequence variation of rRNA genes and the ITS region in *M. major* relative to *M. rubrum*, is equal to or lower than variation among *M. rubrum* variants (**Table 4**), making the distinction of species vs. variant somewhat ambiguous based on these sequences alone. While we are beginning to identify trends in genotype distribution of *M. major/rubrum* ciliates and their association with bloom events, we know little about distinctions in the physiological or behavioral diversity among these variants and if they may possess distinct ecological niches.

While our results strongly suggest that heterotrophic species have less intracladal diversity in marine ecosystems, our samples focused on bloom events and are therefore biased toward the mixotrophic species. Stations where heterotrophic species were abundant, such as two Gulf of Finland (Baltic Sea) samples rich in *M. pupula* (>40% of *Mesodinium* clones), revealed essentially identical sequences within and between these populations. Furthermore, *M. pupula* sequences from the Baltic Sea were nearly identical to those from SGI. In addition, *M. pulex* sequences from SGI and *M. chamaeleon* sequence data from SGI and the Baltic Sea were remarkably similar to strains isolated from Danish waters.

#### Bloom Samples

All temperate and subtropical bloom samples were dominated either by variant B or D from the *M. rubrum/major* complex. The only previous bloom where genetic diversity of *Mesodinium* spp. were assessed was in the Columbia River Estuary, and these populations are also associated with subclade B (Herfort et al., 2011b). Annual blooms of *M. rubrum* occur within the Columbia River, developing first near the mouth of the estuary and later within the main channel in coincidence with neap tides and decreased turbulence (Herfort et al., 2011a). While summer *Mesodinium* populations in the Columbia River are dominated by subclade B, they are largely absent in spring samples, which were instead associated with subclades C and D (Herfort et al., 2011b). Our study only provided “snapshots” of community genetic diversity, and it is likely that diversity at our various sites changes seasonally. Danish cultures of variant F have also been isolated from red-water within islands of the Danish straits (Garcia-Cuetos et al., 2012), however, it is unclear if this clade was primarily responsible for the observed bloom.

Blooms of *M. rubrum* along the North Carolina coastline have been periodically observed (Hathaway, 2014), but no published studies are available. The North Carolina bloom was the only preserved bloom sample that had more than one subclade of *M. rubrum* present, with three other subclades comprising ≤5% of the community. Since the bloom sample was collected near Oregon Inlet, which empties the Pamlico Sound into the Atlantic Ocean, some of the strains present may have been washed in from estuarine populations. The sequences of one of these subclades (G) are identical to that of a strain isolated from the James River estuary in nearby Chesapeake Bay, which would support mixing of estuarine populations into this coastal bloom. The Long Island Sound bloom was also dominated by subclade B (**Figure 2**), and was associated with calm wind speeds (Dierssen et al., 2015). This bloom was detected from the Space Station, using a Hyperspectral Imager for the Coastal Ocean sensor (Dierssen et al., 2015).

Numerous studies have commented on the size variability in natural populations of *M. rubrum*-like ciliates in diverse geographical locations (Taylor et al., 1971; Lindholm, 1981; Crawford, 1993; Rychert, 2004; Montagnes et al., 2008). Garcia-Cuetos et al. (2012) formally described the largest of plastidic *Mesodinium* spp. as *M. major*, which attains cell dimensions of 50 μm × 40 μm (L × W). Particularly large *M. rubrum*-like cells have been observed in many blooms and coastal ocean communities, particularly those associated with upwelling regions (Packard et al., 1978). Both of our *M. major*-dominated bloom samples were from coastal blooms in Brazil and Chile, and were likely associated with upwelling. Both regions have a rich history of documented *Mesodinium* “red tides,” many of which have been described reaching massive proportions. *Mesodinium* blooms have been previously reported in the coastal region of northern São Paulo, Brazil, where our sample was taken, and have been described as forming thin layers of red water 1–2 m below the surface with a maximum thickness of ∼30 cm (Owen et al., 1992). An image of the bloom sampled near Ilha Bela, Brazil, was captured by NASA’s Aqua satellite using the moderate resolution imaging spectrophotometer (MODIS), and the red water formed an offshore patch of staggering proportions, extending 800 km from Rio De Janeiro to south of Florianópolis (Carlowicz et al., 2014). *Mesodinium* blooms in southern Brazil have also been documented to occur near the Itajaí-Açu River (Proença, 2004).

Darwin is thought to have documented the first *Mesodinium* bloom off the Chilean coast while on the H. M. S. Beagle (Darwin, 1906; Hart, 1943), and such occurrences are common in this region during non El Niño periods (Avaria and Muñoz, 1987; Marín et al., 1993). Blooms documented from the Peruvian upwelling zone have reported some of the highest rates of primary production ever measured (Smith and Barber, 1979), and red water patches spanning 100s of square miles (Ryther, 1967). Based on these previous observations and our phylogenetic analysis of the Chilean and Brazilian blooms, it is likely that these recurrent events and perhaps blooms in other upwelling ecosystems are predominantly *M. major*.

#### Non-bloom Samples

*Mesodinium* is abundant in the Baltic Sea (Leppanen and Bruun, 1986) and is known to form occasional blooms in brackish basins around the archipelago region of Åland (Lindholm, 1978). During spring in the northern Baltic Sea, *M. rubrum*-like ciliates dominate ciliate biomass and are thought to account for ∼10% of all primary production (Leppanen and Bruun, 1986). In the lower Baltic Sea, both large and small *M. rubrum*-like ciliates dwell in the upper water column during June, with mostly large cells found at greater depths (Rychert, 2004). Within the Åland region, up to three size classes (∼20, 40, 60 μm) of *M. rubrum*-like ciliates are observed, with the smallest being dominant during autumnal red water events (Lindholm, 1978). Deep (>70 m) layers of pigmented *Mesodinium* have also been found in the Baltic Sea, below the thermocline (Setälä et al., 2005) and near the anoxic boundary of the Gotland Basin (Weber et al., 2014). Our two samples from the Gulf of Finland (GF) revealed a mixed community of *M. rubrum* subclades A, D, and F. While subclade D (*M. major*) is the largest *Mesodinium* spp., both subclades A and F may have a range of cell sizes, even within a single strain (**Table 2**). Surprisingly, the largest component of both GF samples was *M. pupula*, a heterotrophic species that is slightly larger than *M. pulex* (Garcia-Cuetos et al., 2012). The ecology of *M. pupula* is poorly known, but has been shown to be one of the most abundant heterotrophic ciliates in coastal Yellow Sea microbial communities (Jiang et al., 2011). While *M. pulex* has instead been reported as a dominant heterotrophic ciliate species in Baltic communities (Setälä and Kivi, 2003), this species is difficult to discern from *M. pupula* using traditional light microscopy. The near shore station from Hanko, FI, also had subclades D and F, as well as the mixotrophic *M. chamaeleon*, which is capable of possessing a variety of cryptophyte plastid types, including those containing phycocyanin (Moestrup et al., 2012). *M. chameleon* differs from *M. rubrum*-like ciliates, in the structure of its cirrus, its predominantly benthic niche, and in the organization of sequestered cryptophycean organelles (Moestrup et al., 2012). *M. chamaeleon*-like ciliates, reported as green-blue in appearance, have been observed in the Åland region (Lindholm, 1985), as well as in a coastal Rhode Island estuary (Hargraves, 1991). Our Baltic sequence of *M. chamaeleon* was essentially identical to an isolate from coastal Denmark, and both were slightly different from sequences found in SGI.

The CC sample was one of the more surprising due to the unexpected presence of *M. chamaeleon* (13% of clones) in an offshore site. The presence of this “benthic” ciliate in a pelagic environment underscores how little we know about the ecology of this species. The remaining portion of the community was split among variants A, C, and D. Variants C, E, and H are the least distributed and most poorly studied of the *M. major/rubrum* complex, as none have yet been cultured and all have only been found at only one or two sites along the North Pacific coast of the US.

### Cryptophyte Phylogeny

Taxonomic relationships within the cryptophytes, based on traditional morphological and structural traits of their pigments and cell surface, have recently been tested using gene phylogenies (Fitt et al., 2001; Hoef-Emden et al., 2002). These studies have found that while biliprotein type is congruent with molecular phylogenies, the type of inner periplast is not (Hoef-Emden et al., 2002). These phylogenies revealed seven separate lineages within plastid-bearing cryptophytes, all except *Cryptomonas*, containing representatives from marine environments. An additional lineage, represented by the brackish-water species *U. complanatus*, was later discovered (Laza-Martínez, 2012). Previously Hoef-Emden et al. (2005) commented on the potential for accelerated evolutionary rates when constructing *rbcL* phylogenies of cryptophytes, due to shifts in the number of amino acids showing changes in codon usage among early and late diverging taxa. This study was based only on genus *Cryptomonas*, an exclusively freshwater group containing some highly divergent taxa. Yet, despite finding high evolutionary rates for *rbcL*, resulting cryptophyte phylogenies were largely congruent with those of 18S rDNA (Hoef-Emden et al., 2005). Our results, which used the most diverse *rbcL* dataset to date, are also congruent with previously published phylogenies of nuclear 18S rRNA genes (Hoef-Emden et al., 2002). The eight lineages of plastid-bearing cryptophytes were recovered in the *rbcL* phylogeny. Moreover, the *Proteomonas* lineage appeared as the sister group of TPG with high support. While major clades are well-supported in previous phylogenies, the relationships among lineages have not been well-resolved. The clustering of *Proteomonas* and TPG was only partially supported in nucleomorph, but not nuclear, 18S rRNA genes phylogenies in a clade that also included *Guillardia/Hanusia* (Hoef-Emden et al., 2002).

The traditionally used morphological trait of periplast structure, as a continuous sheet or polygonal, has been shown to vary with life stage in the genera *Cryptomonas* and *Proteomonas* and is thus phylogenetically uninformative (Hill and Wetherbee, 1986; Hoef-Emden and Melkonian, 2003). Similar observations of periplast dimorphism have also been made within the *Teleaulax* group (Garcia-Cuetos, 2011), and this trait has traditionally been used to distinguish this genus from *Plagioselmis*. Furthermore, the midventral band, a morphological trait used to distinguish *Teleaulax* (thought to be absent) and *Plagioselmis*, was recently shown to be polyphyletic within this group (Laza-Martínez et al., 2012). The finding of strains with the *T. amphioxeia* and *P. prolonga* morphology sharing nearly identical *rbcL* sequences can be interpreted from the perspective of a dimorphic species, making both names synonym. The *Teleaulax/Plagioselmis* dimorphism can also account for incongruences between microscope-based morphological identifications and environmental DNA sequence identities, as in Bazin et al. (2014), where only *T. acuta* sequences were retrieved from a *P. prolonga* red tide sample. These observations, combined with the polyphyletic phylogeny of *Teleaulax* shown here and previously using 18S rRNA (Deane et al., 2002; Rial et al., 2015), suggest that these genera are in need of taxonomic revision.

### Cryptophyte Diversity and its Role in *Mesodinium* Blooms

**FIGURE 4:**
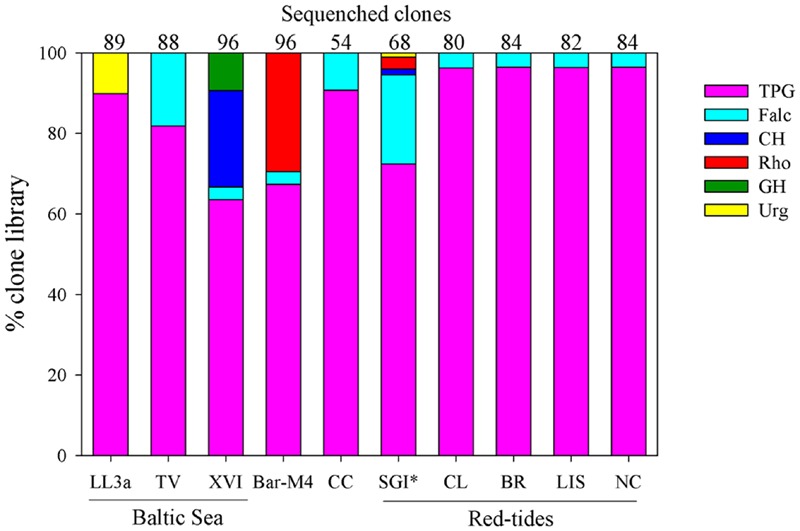
**Community genetic diversity of cryptophyte algal plastids from the Baltic Sea (GF-LL3a, GF-XVI, TV), Barents Sea (Bar-M4), California Current (CC), St George Island (SGI) harbor, coastal Chile (CL), coastal Brazil (BR), Long Island Sound (LIS), and coastal North Carolina (NC).** Community diversity of cryptophytes based on partial sequences of the plastid LSU RuBisCO gene. Categories include the *Teleaulax*/*Plagioselmis*/*Geminigera* (TPG) group, *Falcomonas*-like species (Falc), the *Chroomonas/Hemiselmis* group (CH), *Rhodomonas*-like cryptophytes (Rho), the *Guillardia/Hanusia* group (GH), and *Urgorri*-like cryptophytes (Urg). ^∗^SGI sample was not properly preserved, so the relative proportion sequences within each category may have been compromised.

About half of our samples were from *Mesodinium* blooms, and these samples (excluding SGI) were dominated (>95%) by *T. amphioxeia*, as expected from previous studies (Nishitani et al., 2010, #874; Herfort et al., 2011b, #893). However, clone libraries from non-bloom samples were also largely comprised of TPG sequences, particularly *T. amphioxeia* (**Figure 4**). Exceptions were the high latitude BS and SGI samples, which were largely composed of *T. acuta* and *G. cryophila*-like sequences, respectively. *G. cryophila* appears to primarily be a cold-water species, dominating cryptophyte assemblages in Antarctic Dry Valley Lakes (Bielewicz et al., 2011), and was co-isolated with an Antarctic *M. rubrum* strain (Gustafson et al., 2000). *T. gracilis* and *T. minuta* have also been shown to sustain *in vitro M. rubrum* (clade F) growth (Rial et al., 2015). However, only few sequences of the former and none of the latter were recovered. These results strongly suggest that TPG species may frequently dominate cryptophyte communities in coastal ecosystems, and play critical roles in supporting the productivity of *M. major/rubrum.*

Baltic Sea planktonic communities have been shown to be rich in TPG cryptophytes, and are also known to have *Hemiselmis* and *Rhodomonas* spp. (Hill et al., 1992; Carstensen and Heiskanen, 2007; Hajdu et al., 2007). While none of our Baltic Sea samples were associated with a *Mesodinium* bloom, *Mesodinium* cell counts were not available and thus it is unclear to what degree their plastids may be contaminating the cryptophyte *rbcL* community signal. In addition to TPG species, we also found *Falcomonas, Hemiselmis*, and *U. complanatus* sequences in the Gulf of Finland. *U. companatus* was originally described from French, Spanish, and Portuguese estuaries and has a maximum growth rate at a salinity of 10 psu (Laza-Martínez, 2012); thus its presence within these brackish waters is not surprising. A single clone of *U. complanatus* was also recovered from our BS sample. While the BS was dominated by *T. amphioxeia, M. major* was found in this sample at ∼10 cells/ml, which would equate to about 400–600 *T. amphioxeia* plastids ml^-1^ based on plastid numbers reported by Garcia-Cuetos et al. (2012). Since our community samples were not size fractioned, in this case our clone libraries may have been highly enriched with plastids from this species since it is the preferred prey of the *M. major/rubrum* complex (Yih et al., 2004; Park et al., 2007; Myung et al., 2011; Hansen et al., 2012).

Peaks in cryptophyte abundance have been found to both proceed (Johnson et al., 2013) and co-occur (Kim et al., 2007; Weber et al., 2014) with high levels of *M. rubrum*-like ciliates in coastal ecosystems. However, most historical accounts of *Mesodinium* blooms did not note the abundance or composition of co-occurring cryptophyte communities. While certain aspects of *M. major/rubrum* blooms have been well-studied within a variety of ecosystems, such as the Southampton estuary (Crawford et al., 1997), upwelling zones from Ecuador to Chile (Ryther, 1967; Avaria, 1976; Jimenez and Intriago, 1987), Baltic Sea Fjords (Lindholm, 1978; Lindholm and Mörk, 1990; Sjöqvist and Lindholm, 2011), and portions of the Gulf of California (Gárate-Lizárraga et al., 2002; Bulit et al., 2004), only within the Columbia River estuary (Herfort et al., 2011a) have studies focused on cryptophyte-*Mesodinium* dynamics. Within this system, cryptophytes are high in abundance both prior to and during early *M. rubrum* blooms, but decline as ciliate concentrations increased (Peterson et al., 2013). Observations of *M. rubrum* cells from these communities revealed what appears to be a novel feeding strategy on cryptophyte algae, with numerous prey cells attached to either the cirri and/or feeding tentacles of the ciliates (Peterson et al., 2013). These observations suggest that some *M. rubrum* populations may be able to act opportunistically and quickly consume a surplus of cryptophytes in order to sustain their productivity. In the Columbia River, *M. rubrum* cells from blooms were analyzed for 16S rRNA gene diversity during two consecutive years and found to exclusively possess *T. amphioxeia*-like plastids (Herfort et al., 2011b). In contrast, surface clone libraries from the estuary were nearly devoid of cryptophyte sequences, suggesting they primarily reside at depth (see Peterson et al., 2013).

## Conclusion

Our results solidify the link between *Mesodinium* blooms and *T. amphioxeia-*like cryptophytes in both temperate and subtropical Atlantic and Pacific ecosystems. While multiple variants of the *M. major/rubrum* complex are linked to estuarine and near-shore blooms, our results suggest that variant B is the most common agent in temperate and subtropical regions, while variants A and F may be involved in higher latitude blooms. The largest variant, *M. major* (D), was shown to be associated with shelf-water and offshore blooms in coastal Pacific regions and is likely responsible for the largest and most productive *Mesodinium* blooms (e.g., Ryther, 1967; Packard et al., 1978; Smith and Barber, 1979) associated with upwelling. Even non-bloom samples in our study were dominated by TPG cryptophyte sequences, implicating this group as playing a critical role in marine microbial foodwebs. Their widespread distribution and abundance, may explain why *Mesodinium* ciliates have adapted to exploit them for fueling their acquired phototrophy niche. We recommend that future studies of *Mesodinium* genetic diversity using targeted PCR should use the primers presented here in order to gain further insights into the diversity of the entire genus.

## Author Contributions

MJ contributed to sampling, performed data analysis, and wrote the manuscript. DB performed all of the laboratory work for PCR, cloning, and sequencing preparation and contributed to writing the manuscript. AL-M provided cultures, helped interpret data, and contributed to writing the manuscript. SD, EF, SL, AM, MP, and OS provided field samples and helped edit the manuscript. DS contributed to data interpretation and writing the manuscript.

## Conflict of Interest Statement

The authors declare that the research was conducted in the absence of any commercial or financial relationships that could be construed as a potential conflict of interest.
